# Lateral Entry Fixation Using Three Divergent Pins for Displaced Paediatric Supracondylar Humeral Fractures

**DOI:** 10.5402/2011/137372

**Published:** 2011-09-11

**Authors:** Stephen Paul Guy, Ramakrishna Rao Ponnuru, Sreenadh Gella, Nirmal Tulwa

**Affiliations:** Department of Orthopaedic and Traua Surgery, Pinderfields Hospital, Aberford Road, Wakefield WF1 4DG, UK

## Abstract

*Background*. Supracondylar fractures are the commonest elbow injury in children. Most displaced supracondylar fractures are manipulated and held with a medial/lateral entry or two lateral Kirschner wires. This clinical study has results purely from a three lateral divergent wire technique. *Methods*. Displaced supracondylar fractures were manipulated closed and three lateral divergent wires inserted. Primary study end points were range of movement and carrying angle relative to the contralateral uninjured elbow (Flynn's grading system) and presence of iatrogenic nerve or vessel injury. *Results*. 25 children between 3 and 10 years (median 5, range 3–10) suffered a displaced fracture (15 type III, 10 type IIB). 15 left-, 10 right-sided fractures, 14 boys and 11 girls). 23 were fixed primarily, of these 21 in the first 24 hours. 2 were delayed due to swelling. 2 were fixed secondarily with lateral k-wires after loss of position (from a primarily fixed crossed wire technique). One radial and one median nerve palsy sustained at injury settled. No iatrogenic nerve injuries occurred. 21 Excellent, 3 good and 1 poor result on Flynn's grading. *Conclusions*. The use of three wires on the lateral side in this cohort showed no evidence of slip in fracture position and no iatrogenic nerve injury.

## 1. Introduction

Paediatric supracondylar fractures can be challenging to treat and are the most common elbow fractures in children, accounting for 75% of all paediatric elbow injuries [1, 2]. There are well-known complications associated with supracondylar fractures and their treatment—neurovascular injury, compartment syndrome, and malunion leading to cubitus varus. In displaced fractures the incidence of vascular compromise has been reported between 12% [3] and as high as 19-20% [4, 5]. The amount of neurological complication has ranged between 10% and 20% [4], with the most common nerve palsy being the anterior interosseous nerve [6–8]. The rate of compartment syndrome is estimated to be between 0.1% and 0.3% [9] and in the presence of an ipsilateral forearm fracture can increase to 9% [10]. The occurrence of deformity from malunion varies in the literature; it has been estimated to be 4.2% using data pooled from 1455 patients [11].

Supracondylar fractures are commonly classified according to Gartland [12]. This system was modified by Wilkins to allow for rotational deformity: type I (undisplaced), type IIA (angulated, posterior cortex intact, no rotation), type IIB (angulated, posterior cortex intact, rotational deformity), and type III (displaced with no cortical contact) [13]. Types I and IIA are mainly treated in an above-elbow cast. 

Debate persists in methods of treating displaced supracondylar fractures. The current recommended practice for displaced supracondylar fractures is operative reduction and pin fixation [14]. In practice this varies using two main techniques: two lateral entry wires alone, using a closed pinning technique or a medial and lateral crossed wire technique. On placing medial wires, surgeons may choose to use a closed, miniopen, or open technique depending on their level of experience and current practice. Advocates of the crossed wire technique state this may convey increased biomechanical stability but the ulnar nerve is at risk from the medial wire [15, 16]. With lateral entry only, iatrogenic injury to the ulnar nerve should be avoided but the construct may be biomechanically less stable [17–20].

We designed this study as this is the technique the senior author (N. Tulwa) was taught and teaches his trainees to use. The purpose of this study is to assess if using three closed lateral divergent pins causes iatrogenic nerve injury, deformity (cubitus varus), and poor range of motion. 

## 2. Materials and Methods

The study design was a prospective observational cohort study. Patients were all treated and followed up by the same surgeon (N. Tulwa) in a district general hospital over a period of four years from 2001 to 2005. 25 children (median age 5, age range 3–10) consisting of 14 boys and 11 girls were treated. All consecutive children admitted under the surgeons' on call or referred by colleagues in the same hospital with a type-IIB or type-III displaced fracture were included in the study. 15 fractures were of type III, and 10 were of type IIB; none were open. 15 were left-sided and 10 were right-sided injuries. Preoperative neurovascular deficits were noted.

General anaesthetic was used in all cases. One dose of intravenous cefuroxime was given on induction, appropriate for their weight. A standardised method was used by N. Tulwa—A high-arm tourniquet was placed on the arm, but not inflated, should the rare event occur that the fracture site required to be opened. The limb was prepared and draped. Reduction can be considered a “standard” technique by many—this involved manual traction for 2 minutes with the elbow flexed at 20 degrees, controlling rotation of the fracture by the medial and lateral humeral epicondyles. The forearm was then pronated, as this controls the medial rotation, and with flexion locks the fracture in place. This technique was adequate for reduction in all the patients of the study. The reduction was then imaged in anteroposterior, lateral and two oblique planes to observe the medial and lateral columns. When acceptable reduction was observed, the arm was held in maximal flexion by wrapping a crepe bandage around the forearm and arm. This freed up the surgical assistant and allowed for better rotational control of the fracture because the forearm and upper arm would now only rotate together as one unit. 

**Figure 1:**
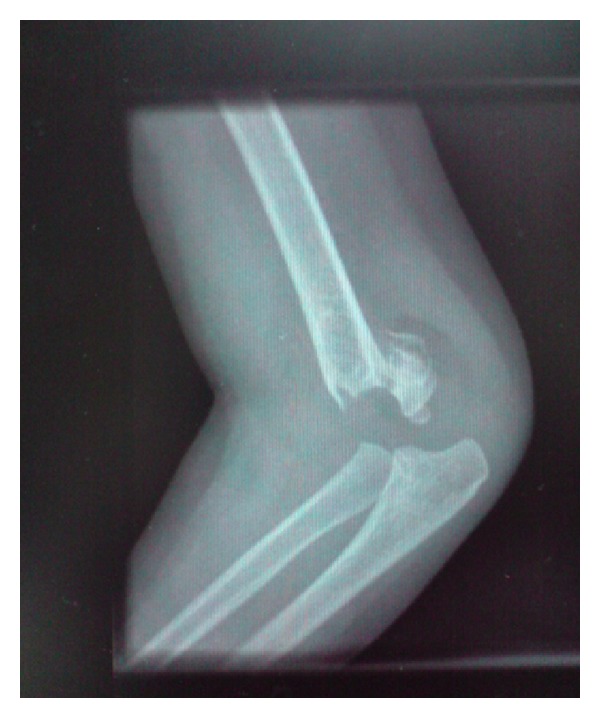
Preoperative lateral radiograph.

**Figure 2:**
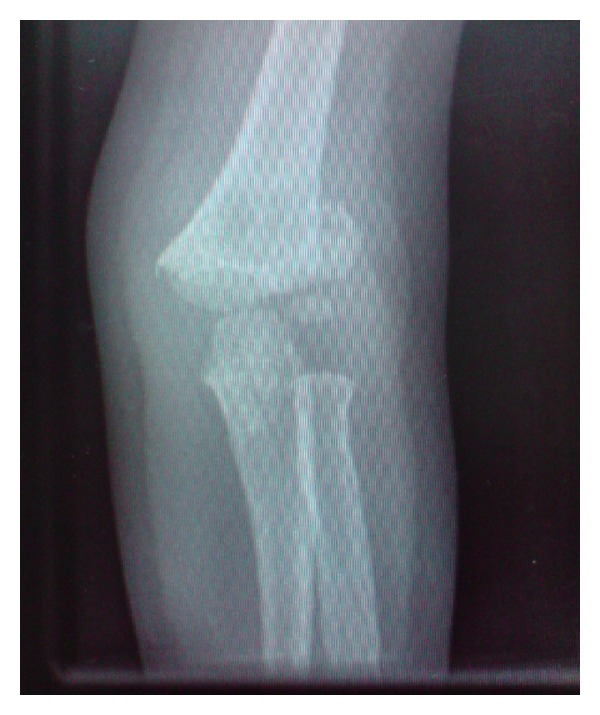
Preoperative AP radiograph.

**Figure 3:**
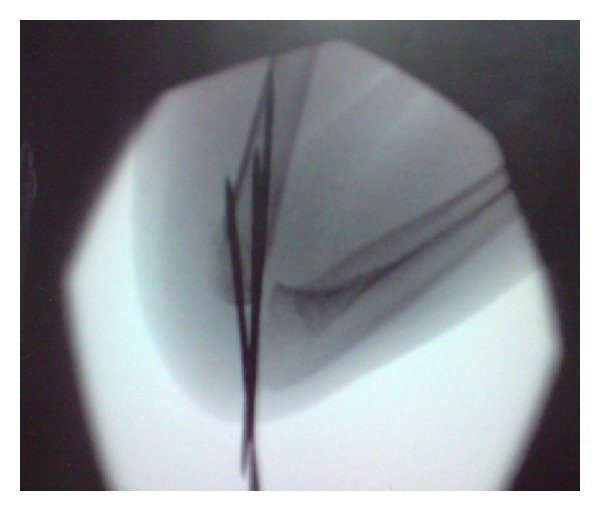
Intraoperative lateral Image.

**Figure 4:**
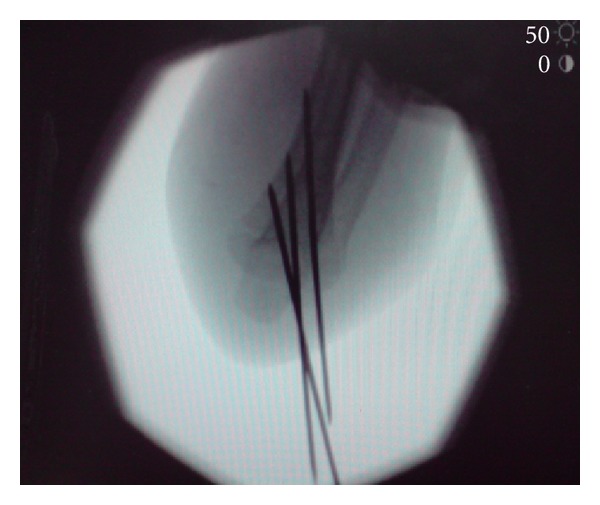
Intraoperative flexed AP Image.

Three Kirschner wires (1.6 mm) were then inserted from the lateral side. Placement of the divergent wires was crucial for stability and commenced with the most lateral wire into the lateral column at the fracture site. The next wire inserted was divergent and entered the medial column with maximal pin separation as was possible at the fracture site. With the two wires now as a reference, the third wire was inserted into the middle column. On the lateral intraoperative radiograph you will see that the pins incline in the anteroposterior direction in accordance with the normal bony anatomy of the distal humerus (Figures 1, 2, 3, 4). Fracture stability was assessed by screening the fracture under varus/valgus, flexion/extension and rotational stresses. The wires were then bent and cut outside the skin, and the limb was immobilised in an above-elbow slab with the elbow at seventy degrees. Patients were all discharged within 24 hours of surgery.

Patients were followed up postoperatively at one week, three weeks (with removal of Kirschner wires and above-elbow cast), six weeks, and multiples of six weeks until they regained full range of movement or no further improvement was noted. If patients had neurovascular problems, they were also followed up until the symptoms resolved. Check X-rays were taken to assess union alone, and no contralateral elbow X-rays were taken as a comparator. As the X-rays were not standardised, we have not included measurement of Baumann's angle due to poor reliability. Thus the primary outcomes measured are purely clinical. We have used the Flynn's grading system—namely, the difference in carrying angle (cosmetic factor) and range of movement (functional factor), compared to the uninjured elbow [21]. The overall grading is based on the worst functional or cosmetic factor.

## 3. Results

23 fractures were primarily fixed by NT; 2 were secondarily fixed, having been referred by consultant colleagues in the same hospital when their previous crossed wire fixation had slipped. Both these cases had failed at the one-week check film and were due to poor surgical technique. The referred cases were both revised to lateral wires at 10 days after injury. Of the 23 primarily fixed fractures, 21 were done within 24 hours. 2 were operated upon at day 4 and day 7 due to gross swelling and maintained in traction and elevation until then. Two children had neurological injury on presentation (both type III), one radial and one anterior interosseous nerve palsy. Both recovered between the 6- and 12-week clinics. One presented with an isolated brachial artery injury that was explored and repaired with a vein patch by a consultant vascular surgeon with no vascular sequelae. No elbows were opened for fracture fixation. No wires became loose, and none lost position. One patient had one superficial pin site infection treated uneventfully with five-day oral flucloxacillin. All had a minimum followup of 12 weeks (mean 14.6 weeks, range 12–48 weeks).

Using Flynn's grading system 24 out of 25 patients achieved a satisfactory result. 21 patients had an excellent outcome rating (including one of the two patient's revised from crossed wires, one patient operated on day 4 and the patient with the anterior interosseous nerve palsy), 3 achieved a good rating, due to loss of movement alone (including one of the two patient's revised from crossed wires, the one patient who had the vascular repair and the patient with the radial nerve palsy), and 1 achieved a poor rating, again due to loss of movement alone (delayed presentation due to neglect over 48 hours after injury, grossly swollen and treated with traction and elevation for a week) (Table 1). No loss of carrying angle or varus deformity was noted clinically. All fractures united.

## 4. Discussion

The aims in the management of the displaced supracondylar fracture are to reduce and immobilise the fracture whilst reducing morbidity. The two main issues are iatrogenic nerve injury and loss of reduction leading to malunion with poor cosmetic and functional outcome. The main debated methods of holding the fracture in the literature are crossed medial and lateral entry wires or two lateral entry wires alone. 

A proposed benefit of using crossed wires is increasing the stability of the fracture fixation thus decreasing the potential for loss of reduction. This is at the possible increased risk of iatrogenic ulnar nerve injury. Advocates of the lateral wire technique will cite the avoidance of iatrogenic ulnar nerve injury at the expense of a less biomechanically stable construct.

Biomechanical studies suggest that crossed wires provide greater torsional stability [17–20]. The strength using crossed wires can be further improved by increasing the number of wires and divergence of the wires in the distal humerus [20]. In lateral wire fixation, divergent wires have been shown to be more stable in extension and varus loading than crossed wires but not in valgus [20]. There are reports of clinical failures of laterally placed wires, thought to be due to poor technique in reduction and fixation [22]. Reports vary as to the loss of reduction using lateral wires. In the systematic review by Brauer et al., they observed that the probability of deformity, from loss of position, was 0.58 times lower with medial/lateral crossed wires than with lateral entry wires [11].

Studies have shown an increased incidence of iatrogenic nerve injury when a medial wire is used. Skaggs et al. observed no loss of reduction when comparing two groups using crossed wires and lateral wires. There was an increased incidence of iatrogenic nerve injury in 17 out of 160 (10.6%) cases treated with a medial wire [16]. Data pooled from 1455 patients found that the incidence of ulnar nerve iatrogenic injury was 5.04 times higher in medial/lateral wire fixation compared to lateral entry fixation [11]. There is also concern about delayed iatrogenic nerve injury using medial wires [23].

The lateral technique in the literature is itself not without iatrogenic nerve problems. Foead et al. recognised no loss of position comparing medial/lateral pin fixation to lateral pin fixation [24]. 5 out of 34 (14.7%) in the medial placed wire group sustained iatrogenic ulnar nerve palsy, and 3 out of 32 (9.4%) of the lateral entry group sustained an iatrogenic nerve injury (two ulnar and one radial nerve). Likewise Shamsuddin et al. found no loss in reduction between medial/lateral pin fixation and lateral pin fixation groups but noted 2 iatrogenic ulnar nerve palsies in the medial/lateral group and 2 iatrogenic nerve palsies (one median and one radial nerve) in the lateral entry group [25].

Recently, Kocher et al. have shown there is no statistical difference between medial/lateral wire entry and lateral entry in terms of loss of position in a study with sufficient power to detect 10% difference between the two groups [26]. To attain statistical significance analysing iatrogenic nerve injury is more difficult. It has been suggested that to show a difference in iatrogenic nerve injury between medial/lateral entry crossed wires and lateral entry wires in a suitably powered study with an *α* of 0.05 and a *β* of 0.20 (power 80%) would need patient arms of approximately 2000. This study may never prove to be practical [11].

The senior author would like to highlight that a strength of the study is the easily taught and reproducible technique of fixation. This has implications for trainees and indeed fixation of these difficult fractures amongst nonspecialised trauma surgeons. The routine use of three wires has firm biomechanical evidence already highlighted in the text.

Weaknesses of the paper are the heterogeneous group of patients. We decided to include the secondarily fixed patients to highlight that fractures do displace and surgeons need timely skills to deal with this unfortunate, rare eventuality. It would appear, however, anecdotally in our paper, that a poorer outcome may be related to delay in definitive fixation (delay in presentation/failure of a previous fixation) or other traumatic insult (vascular injury or nerve neuropraxia). The technique using 3 wires has the potential increased probability of iatrogenic nerve injury, pin track infection and the technical difficulty of getting three wires in a relatively small area. To address this, studies have recommended the use only of a third wire when the fracture remains unstable after two lateral wires have been used [22, 27]. Bias of outcome could also occur because the Flynn grading was documented by the operating surgeon and that the surgeon has a paediatric speciality interest and is experienced in the management of these fractures. 

In this study there were no iatrogenic nerve injuries, none of the fractures lost carrying angle, and only one difficult case had an unsatisfactory result according to Flynn's criteria due to poor postoperative range of motion compared to the contralateral elbow.

## Figures and Tables

**Table 1:** Definition of Flynn's grading system and patient outcomes.

Result	Flynn's Rating	Cosmetic Factor—loss of carrying angle (degrees)	Outcome of patients in study	Functional Factor—loss of movement (degrees)	Outcome of patients in study
Satisfactory	Excellent	0–5	25	0–5	21
Satisfactory	Good	6–10	0	6–10	3^#^
Satisfactory	Fair	11–15	0	11–15	0
Unsatisfactory	Poor	>15	0	>15	1*

^#^Includes a case with a vascular repair, a patient with a secondarily fixed fracture, and a patient with a radial nerve palsy.

*This is the case with delayed presentation due to neglect.
